# Thio­phene-2-carbaldehyde azine

**DOI:** 10.1107/S1600536813013275

**Published:** 2013-05-18

**Authors:** David K. Geiger, H. Cristina Geiger, Laura M. Szczesniak

**Affiliations:** a Department of Chemistry, State University of New York-College at Geneseo, 1 College Circle, Geneseo, NY 14454, USA

## Abstract

The asymmetric unit of the title compound, C_10_H_8_N_2_S_2_, is composed of two independent half-mol­ecules, each residing on a center of symmetry. In the crystal, weak C—H⋯π inter­actions join the two symmetry-independent molecules together into interlinked chains parallel to [011]. The crystal structure was refined as a two-component pseudo-merohedral twin using the twin law 001 0-10 100. The refined domain fractions are 0.516 (3) and 0.484 (3).

## Related literature

For the structure of pyridine-4-carbaldehyde, see: Shanmuga Sundara Raj *et al.* (2000 ▶) and for the structure of (*E*)-1-di­phenyl­methyl­idene-2-[(1*H*-indol-3-yl)methyl­idene]hydrazine, see: Archana *et al.* (2010 ▶).
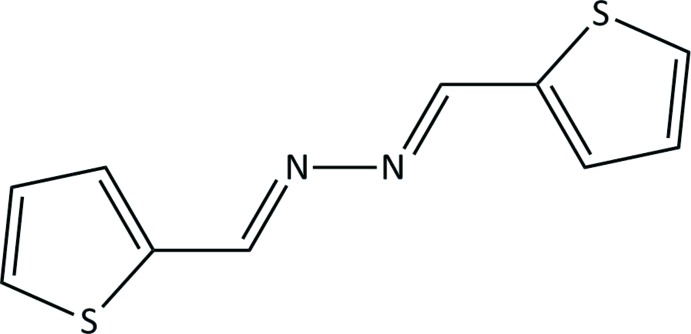


## Experimental

### 

#### Crystal data

C_10_H_8_N_2_S_2_
*M* *_r_* = 220.30
Monoclinic, 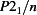

*a* = 9.681 (2) Å
*b* = 11.399 (3) Å
*c* = 9.694 (2) Å
β = 100.850 (9)°
*V* = 1050.6 (5) Å^3^
*Z* = 4
Mo *K*α radiation
μ = 0.47 mm^−1^
*T* = 200 K
0.50 × 0.20 × 0.20 mm

#### Data collection

Bruker SMART X2S CCD diffractometer
Absorption correction: multi-scan (*SADABS*; Bruker, 2010 ▶) *T*_min_ = 0.69, *T*_max_ = 0.91
7000 measured reflections
1890 independent reflections
1349 reflections with *I* > 2σ(*I*)
*R*_int_ = 0.073

#### Refinement

*R*[*F* ^2^ > 2σ(*F* ^2^)] = 0.059
*wR*(*F* ^2^) = 0.156
*S* = 0.99
1890 reflections
128 parameters
H-atom parameters constrained
Δρ_max_ = 0.61 e Å^−3^
Δρ_min_ = −0.28 e Å^−3^

### 

Data collection: *APEX2* (Bruker, 2010 ▶); cell refinement: *SAINT* (Bruker, 2010 ▶); data reduction: *SAINT*; program(s) used to solve structure: *SHELXS97* (Sheldrick, 2008 ▶); program(s) used to refine structure: *SHELXL97* (Sheldrick, 2008 ▶); molecular graphics: *PLATON* (Spek, 2009 ▶) and *Mercury* (Macrae *et al.*, 2008 ▶); software used to prepare material for publication: *publCIF* (Westrip, 2010 ▶).

## Supplementary Material

Crystal structure: contains datablock(s) global, I. DOI: 10.1107/S1600536813013275/zp2003sup1.cif

Structure factors: contains datablock(s) I. DOI: 10.1107/S1600536813013275/zp2003Isup2.hkl

Supplementary material file. DOI: 10.1107/S1600536813013275/zp2003Isup3.mol

Supplementary material file. DOI: 10.1107/S1600536813013275/zp2003Isup4.cml

Additional supplementary materials:  crystallographic information; 3D view; checkCIF report

## Figures and Tables

**Table 1:** Hydrogen-bond geometry (Å, °)

*D*—H⋯*A*	*D*—H	H⋯*A*	*D*⋯*A*	*D*—H⋯*A*
C1—H1⋯C8	0.95	2.77	3.683 (7)	161
C1—H1⋯C9	0.95	2.85	3.576 (7)	134
C8—H8⋯C4^i^	0.95	2.77	3.663 (7)	156

Symmetry code: (i) 
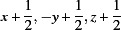
.

**Fig. 1.**
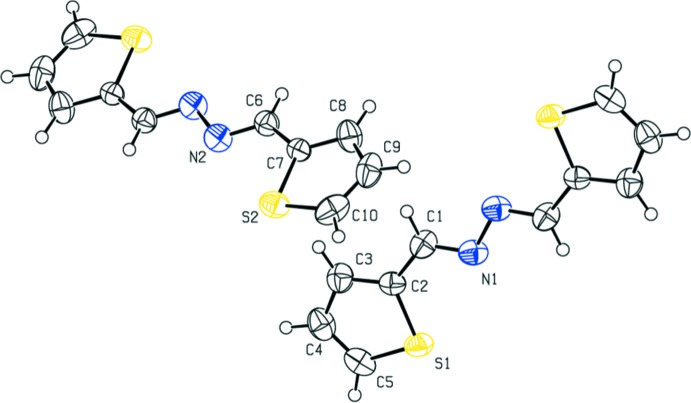
Perspective view of the title compound showing the atom-labeling scheme used for the two half molecules in the asymmetric unit. Displacement ellipsoids of the nonhydrogen atoms are drawn at the 50% probability level.

**Fig. 2.**
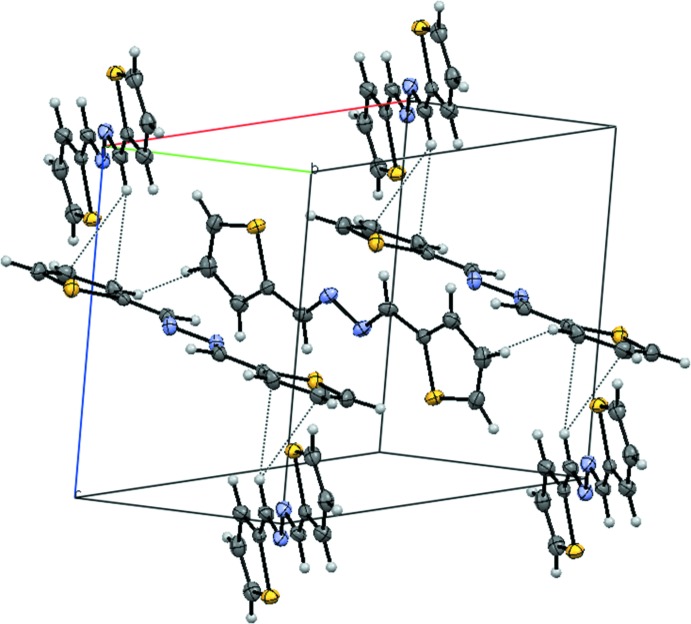
The unit cell of the title compound showing the linked chains parallel to [011].

## supplementary crystallographic information

### Comment

The title compound was a side product in the attempted reduction of a nitro-substituted benzimidazole derivative but was subsequently rationally synthesized as outlined in the *experimental* section.

Thiophene-2-carbaldehyde azine crystallizes with two half-molecules in the asymmetric unit. Each sits on a crystallographically required center of symmetry. Figure 1 shows a perspective view of the two molecules with the atom-labeling scheme. The hydrazine substitutents adopt a (1E,2E) configuration, as required by the crystallographically imposed symmetry. The two molecules are essentially planar. The thiophene containing S1 is canted 1.63 (12)° from the molecular plane and the thiophene containing S2 is canted 1.63 (15)° from the molecular plane. The N1—C1—C2—S2 and N2—C6—C7—S2 torsional angles are 0.4 (6)° and 1.8 (7)°, respectively, and the N1'-N1—C1—C2 and N2'-N2—C6—C7 torsional angles are 178.3 (4)° and 178.9 (4)°, respectively. The orientation of the substituents is similar to that found for pyridine-4-carbaldehyde azine, 1.12 (9)°, (Shanmuga Sundara Raj *et al.*, 2000) and the indole ring in (*E*)-1-diphenylmethylidene-2-[(1*H*-indol-3-yl)methylidene]hydrazine, 0.95 (10)°, (Archana *et al.*, 2010).

A view of the unit cell is shown in Figure 2. Weak C—H···π interactions between the symmetry independent molecules result in chains parallel to [011]. The C1—H1···C8 and C1—H1···C9 intermolecular distances are 2.77 and 2.85 Å, respectively. There is a short intermolecular distance of C8—H8···C4 (2.77 Å).

### Experimental

0.126 ml (4.05 mmol) of hydrazine hydrate in 1 ml of ethanol was slowly added to 0.82 ml (0.98 g, 8.8 mmol) of 2-thiophene carboxaldehyde in 35 ml of ethanol at room temperature with stirring. The reaction was refluxed for 4 h and monitored by TLC. The reaction mixture was cooled to 0°C and the product was obtained by vacuum filtration. 0.32 g obtained (33% yield). *R*_f_ = 0.88 (EtOAc/EtOH, 2:1 (*v*/*v*) on silica gel). mp = 147–149°C. ^1^H NMR (400 MHz, CDCl_3_): δ, 8.80 (s, 2H); 7.50 (d, 2H); 7.44 (d, 2H); 7.14 (dd, 2H). ^13^C NMR (CDCl_3_): δ, 155.80, 139.00, 132.50, 130.04, 127.84.

Single crystals suitable for X-ray diffraction were obtained by slow evaporation of an ethanol/ethylacetate solution.

### Refinement

The autosolve routine of the APEXII software (Bruker, 2010) chose an orthorhombic, *C*-centered cell, but no suitable space group could be found. Subsequently, the structure was solved in *P*2_1_/*n* and refined to *R*_1_ = 0.17 and *S* = 3.27. Refinement as a pseudo-merohedral twin with the twin law 001 0–10 100 resulted in a dramatic improvement in the model. The domain fractions refined to 0.516 (3) and 0.484 (3).

The H atoms were refined using a riding model with a C—H distance of 0.95 Å and the thermal parameters were set using the approximation *U*_iso_ = 1.2*U*_eq_(C).

### Crystal data

**Table:**

C_10_H_8_N_2_S_2_	*D*_x_ = 1.393 Mg m^−^^3^
*M**_r_* = 220.30	Melting point: 420 K
Monoclinic, *P*2_1_/*n*	Mo *K*α radiation, λ = 0.71073 Å
*a* = 9.681 (2) Å	Cell parameters from 1222 reflections
*b* = 11.399 (3) Å	θ = 2.8–21.5°
*c* = 9.694 (2) Å	µ = 0.47 mm^−^^1^
β = 100.850 (9)°	*T* = 200 K
*V* = 1050.6 (5) Å^3^	Prism, yellow
*Z* = 4	0.50 × 0.20 × 0.20 mm
*F*(000) = 456	

### Data collection

**Table:**

Bruker SMART X2S CCD diffractometer	1890 independent reflections
Radiation source: XOS X-beam microfocus source	1349 reflections with *I* > 2σ(*I*)
Doubly curved silicon crystal monochromator	*R*_int_ = 0.073
Detector resolution: 8.3330 pixels mm^-1^	θ_max_ = 25.4°, θ_min_ = 1.8°
ω scans	*h* = −11→11
Absorption correction: multi-scan (*SADABS*; Bruker, 2010)	*k* = −12→13
*T*_min_ = 0.69, *T*_max_ = 0.91	*l* = −11→11
7000 measured reflections	

### Refinement

**Table:**

Refinement on *F*^2^	Primary atom site location: structure-invariant direct methods
Least-squares matrix: full	Secondary atom site location: difference Fourier map
*R*[*F*^2^ > 2σ(*F*^2^)] = 0.059	Hydrogen site location: inferred from neighbouring sites
*wR*(*F*^2^) = 0.156	H-atom parameters constrained
*S* = 0.99	*w* = 1/[σ^2^(*F*_o_^2^) + (0.0825*P*)^2^] where *P* = (*F*_o_^2^ + 2*F*_c_^2^)/3
1890 reflections	(Δ/σ)_max_ < 0.001
128 parameters	Δρ_max_ = 0.61 e Å^−^^3^
0 restraints	Δρ_min_ = −0.28 e Å^−^^3^

### Special details

**Table:**

Geometry. All e.s.d.'s (except the e.s.d. in the dihedral angle between two l.s. planes) are estimated using the full covariance matrix. The cell e.s.d.'s are taken into account individually in the estimation of e.s.d.'s in distances, angles and torsion angles; correlations between e.s.d.'s in cell parameters are only used when they are defined by crystal symmetry. An approximate (isotropic) treatment of cell e.s.d.'s is used for estimating e.s.d.'s involving l.s. planes.
Refinement. Refinement of *F*^2^ against ALL reflections. The weighted *R*-factor *wR* and goodness of fit *S* are based on *F*^2^, conventional *R*-factors *R* are based on *F*, with *F* set to zero for negative *F*^2^. The threshold expression of *F*^2^ > σ(*F*^2^) is used only for calculating *R*-factors(gt) *etc*. and is not relevant to the choice of reflections for refinement. *R*-factors based on *F*^2^ are statistically about twice as large as those based on *F*, and *R*- factors based on ALL data will be even larger.

### Fractional atomic coordinates and isotropic or equivalent isotropic displacement parameters (Å^2^)

**Table:**

	*x*	*y*	*z*	*U*_iso_*/*U*_eq_	
S1	−0.15035 (15)	0.24362 (18)	−0.24483 (16)	0.0462 (4)	
S2	−0.25404 (15)	0.26513 (11)	0.35231 (15)	0.0486 (5)	
N1	−0.0295 (4)	0.0438 (3)	−0.0474 (4)	0.0442 (12)	
N2	−0.0495 (4)	0.4567 (3)	0.4718 (4)	0.0446 (13)	
C1	−0.0425 (5)	0.1418 (4)	0.0133 (5)	0.0432 (14)	
H1	−0.0153	0.147	0.1125	0.052*	
C2	−0.0969 (5)	0.2439 (3)	−0.0643 (6)	0.0361 (13)	
C3	−0.1093 (6)	0.3536 (4)	−0.0119 (6)	0.0484 (14)	
H3	−0.084	0.3726	0.085	0.058*	
C4	−0.1644 (5)	0.4370 (4)	−0.1182 (6)	0.0540 (15)	
H4	−0.1815	0.5171	−0.1002	0.065*	
C5	−0.1889 (6)	0.3885 (4)	−0.2458 (6)	0.0519 (15)	
H5	−0.2244	0.4312	−0.3291	0.062*	
C6	0.0080 (5)	0.3572 (4)	0.4585 (5)	0.0395 (13)	
H6	0.1073	0.35	0.4843	0.047*	
C7	−0.0737 (5)	0.2566 (4)	0.4056 (5)	0.0340 (13)	
C8	−0.0274 (6)	0.1447 (4)	0.3957 (6)	0.0505 (15)	
H8	0.0683	0.122	0.4225	0.061*	
C9	−0.1364 (6)	0.0664 (4)	0.3416 (5)	0.0572 (16)	
H9	−0.1225	−0.0147	0.3265	0.069*	
C10	−0.2655 (6)	0.1210 (4)	0.3131 (6)	0.0570 (16)	
H10	−0.3511	0.082	0.2757	0.068*	

### Atomic displacement parameters (Å^2^)

**Table:**

	*U*^11^	*U*^22^	*U*^33^	*U*^12^	*U*^13^	*U*^23^
S1	0.0523 (12)	0.0503 (7)	0.0336 (10)	−0.0004 (5)	0.0017 (7)	0.0045 (5)
S2	0.0389 (11)	0.0509 (7)	0.0543 (13)	−0.0039 (6)	0.0041 (7)	0.0047 (6)
N1	0.048 (3)	0.039 (2)	0.043 (3)	−0.0068 (19)	0.002 (2)	0.0083 (19)
N2	0.040 (3)	0.040 (2)	0.053 (3)	−0.0068 (17)	0.009 (2)	0.0027 (19)
C1	0.055 (4)	0.039 (2)	0.037 (3)	−0.008 (2)	0.015 (3)	0.003 (2)
C2	0.028 (3)	0.044 (2)	0.037 (3)	−0.0046 (19)	0.007 (2)	0.004 (2)
C3	0.055 (4)	0.045 (2)	0.047 (3)	0.000 (3)	0.017 (3)	0.001 (3)
C4	0.047 (4)	0.041 (3)	0.075 (4)	−0.001 (2)	0.016 (3)	0.002 (3)
C5	0.041 (3)	0.053 (3)	0.061 (4)	0.004 (2)	0.007 (3)	0.015 (3)
C6	0.037 (3)	0.042 (3)	0.041 (3)	0.001 (2)	0.010 (2)	0.001 (2)
C7	0.030 (3)	0.041 (2)	0.032 (3)	0.0031 (19)	0.010 (2)	0.005 (2)
C8	0.045 (3)	0.046 (3)	0.065 (4)	0.002 (2)	0.022 (3)	−0.002 (3)
C9	0.083 (5)	0.040 (3)	0.054 (4)	−0.004 (3)	0.026 (3)	−0.004 (2)
C10	0.071 (4)	0.051 (3)	0.048 (4)	−0.027 (3)	0.007 (3)	0.000 (3)

### Geometric parameters (Å, º)

**Table:**

S1—C5	1.693 (5)	C3—H3	0.95
S1—C2	1.729 (6)	C4—C5	1.335 (7)
S2—C10	1.685 (5)	C4—H4	0.95
S2—C7	1.728 (5)	C5—H5	0.95
N1—C1	1.280 (5)	C6—C7	1.432 (6)
N1—N1^i^	1.402 (7)	C6—H6	0.95
N2—C6	1.281 (5)	C7—C8	1.361 (6)
N2—N2^ii^	1.412 (7)	C8—C9	1.406 (7)
C1—C2	1.431 (6)	C8—H8	0.95
C1—H1	0.95	C9—C10	1.377 (7)
C2—C3	1.364 (6)	C9—H9	0.95
C3—C4	1.429 (7)	C10—H10	0.95
			
C5—S1—C2	91.6 (3)	C4—C5—H5	123.3
C10—S2—C7	91.9 (3)	S1—C5—H5	123.3
C1—N1—N1^i^	112.6 (5)	N2—C6—C7	121.6 (5)
C6—N2—N2^ii^	112.5 (5)	N2—C6—H6	119.2
N1—C1—C2	121.8 (5)	C7—C6—H6	119.2
N1—C1—H1	119.1	C8—C7—C6	127.5 (5)
C2—C1—H1	119.1	C8—C7—S2	111.1 (4)
C3—C2—C1	126.8 (5)	C6—C7—S2	121.4 (3)
C3—C2—S1	110.4 (4)	C7—C8—C9	112.8 (5)
C1—C2—S1	122.7 (3)	C7—C8—H8	123.6
C2—C3—C4	112.9 (5)	C9—C8—H8	123.6
C2—C3—H3	123.6	C10—C9—C8	112.2 (5)
C4—C3—H3	123.6	C10—C9—H9	123.9
C5—C4—C3	111.7 (5)	C8—C9—H9	123.9
C5—C4—H4	124.1	C9—C10—S2	112.1 (4)
C3—C4—H4	124.1	C9—C10—H10	124.0
C4—C5—S1	113.5 (4)	S2—C10—H10	124.0

Symmetry codes: (i) −*x*, −*y*, −*z*; (ii) −*x*, −*y*+1, −*z*+1.

### Hydrogen-bond geometry (Å, º)

**Table:**

*D*—H···*A*	*D*—H	H···*A*	*D*···*A*	*D*—H···*A*
C1—H1···C8	0.95	2.77	3.683 (7)	161
C1—H1···C9	0.95	2.85	3.576 (7)	134
C8—H8···C4^iii^	0.95	2.77	3.663 (7)	156

Symmetry code: (iii) *x*+1/2, −*y*+1/2, *z*+1/2.
